# Efficacy of an Enhanced Implementation Strategy to Increase Parent Engagement with a Health Promotion Program in Childcare

**DOI:** 10.3390/ijerph19010106

**Published:** 2021-12-23

**Authors:** Courtney T. Luecking, Cody D. Neshteruk, Stephanie Mazzucca, Dianne S. Ward

**Affiliations:** 1 Department of Dietetics and Human Nutrition, College of Agriculture, Food and Environment, University of Kentucky, Lexington, KY 40506, USA; 2 Department of Population Health Sciences, Duke University School of Medicine, Durham, NC 27710, USA; cody.neshteruk@duke.edu; 3 Brown School, Washington University in St. Louis, St. Louis, MO 63130, USA; smazzucca@wustl.edu; 4 Center for Health Promotion and Disease Prevention, Department of Nutrition, University of North Carolina at Chapel Hill, Chapel Hill, NC 27599, USA; dsward@email.unc.edu

**Keywords:** implementation, nutrition, physical activity, early care and education, family

## Abstract

Previous efforts to involve parents in implementation of childcare-based health promotion interventions have yielded limited success, suggesting a need for different implementation strategies. This study evaluated the efficacy of an enhanced implementation strategy to increase parent engagement with *Healthy Me*, *Healthy We*. This quasi-experimental study included childcare centers from the second of two waves of a cluster-randomized trial. The standard approach (giving parents intervention materials, prompting participation at home, inviting participation with classroom events) was delivered in 2016–2017 (29 centers, 116 providers, and 199 parents). The enhanced approach (standard plus seeking feedback, identifying and addressing barriers to parent participation) was delivered in 2017–2018 (13 centers, 57 providers, and 114 parents). Parent engagement was evaluated at two levels. For the center-level, structured interview questions with providers throughout the intervention were systematically scored. For the parent-level, parents completed surveys following the intervention. Differences in parent engagement were evaluated using linear regression (center-level) and mixed effects (parent-level) models. Statistical significance was set at *p <* 0.025 for two primary outcomes. There was no difference in parent engagement between approaches at the center-level, *β* = −1.45 (95% confidence interval, −4.76 to 1.87), *p* = 0.38l. However, the enhanced approach had higher parent-level scores, *β* = 3.60, (95% confidence interval, 1.49 to 5.75), *p* < 0.001. In the enhanced approach group, providers consistently reported greater satisfaction with the intervention than parents (*p* < 0.001), yet their fidelity of implementing the enhanced approach was low (less than 20%). Results show promise that parent engagement with childcare-based health promotion innovations can positively respond to appropriately designed and executed implementation strategies, but strategies need to be feasible and acceptable for all stakeholders.

## 1. Introduction

Early care and education (ECE) settings are important for improving children’s dietary and physical activity behaviors [1]. A variety of policies, programs, and practices (i.e., innovations) within ECE settings have shown, under carefully controlled research conditions, to positively influence children’s diet and physical activity [2]. However, low adoption and/or insufficient implementation of these evidence-based innovations has yielded mixed effects in more pragmatic conditions [3,4]. In order to produce sustained positive change in young children’s dietary and physical activity behaviors, strategies are needed to facilitate uptake and enhance implementation of effective innovations in ECE in real world conditions [5,6].

Commonly used implementation strategies for supporting evidence-based innovations that promote healthy eating and physical activity in ECE settings include educational meetings and materials for ECE providers, educational outreach visits or academic detailing (in-person visits to ECE setting to provide information and support), small incentives, audit and feedback, opinion leaders, and reminders and tailored interventions [7,8]. Although these implementation strategies likely improve implementation of innovations in ECE settings, they do not appear to impact children’s dietary and physical activity behaviors [8]. This discrepancy between implementation and children’s behaviors suggests a need for additional or different implementation strategies to enhance the effectiveness of existing innovations in real world settings. Implementation strategies focused on all caregivers, including parents, have had limited utilization but may address this discrepancy.

Involving parents in the implementation of ECE center-based innovations can strengthen intervention effects on eating, physical activity, and/or obesity prevention outcomes [9]. Some innovations have tested collaborative approaches with parents like goal setting or family events, but most have used low-intensity, passive approaches such as sending home brochures or worksheets that prompt minimal parent engagement [9,10]. To improve the effectiveness of innovations, interactions among caregivers need to be bidirectional [11]. Intentional selection of strategies that incorporate behavior change techniques associated with effective interventions (e.g., goal setting, barrier identification, or problem solving) could support bidirectional interactions [12]. Furthermore, while many innovations aim to involve parents, few have measured and reported parent engagement [10]. Prior studies have measured parent engagement as either program enrollment or attendance [13,14], failing to capture the dynamic, multi-faceted process between ECE providers and parents that truly defines parent engagement [15]. *Healthy Me*, *Healthy We* (HMHW) is one example of an innovation designed to promote parent engagement with efforts to support healthier eating and physical activity for 3–4-year-old children attending ECE centers [16,17,18]. This 8-month intervention included a set of implementation strategies for ECE providers to support parents in using components of HMHW at home, but parents reported low levels of implementation support and subsequently low levels of parent engagement [19]. As such, questions remain about the effectiveness of explicit strategies to involve parents in ways that improve implementation of innovations [10].

Based on the limited success of previous studies, including that of HMHW, research is needed to identify strategies that facilitate collaborative efforts between ECE providers and parents to enable parents to be involved with implementation of innovations that support healthier behaviors for young children attending child care [6]. Therefore, the primary aim of this study was to evaluate the efficacy of an enhanced implementation strategy, compared to standard implementation, to increase parent engagement with the HMHW intervention. We hypothesized that ECE centers using the enhanced implementation strategy would have a higher parent engagement score at the end of the intervention period compared to ECE centers that used the standard implementation strategy. A secondary aim of this study was to evaluate implementation outcomes (i.e., acceptability, feasibility, appropriateness, and fidelity) [20] for the enhanced implementation approach.

## 2. Materials and Methods

This study used a post-only with nonequivalent control group quasi-experimental study design [21] embedded within the cluster randomized controlled trial of HMHW (Clinical Trials ID: NCT 02330354) [17]. Access to a delayed intervention control group from the randomized trial offered an important opportunity to better understand the successes and failures of the implementation approach and further explore how to increase parent involvement with health promotion programs [5]. The Institutional Review Board at the University of North Carolina at Chapel Hill approved all protocols. Results from this study are reported according to the Standards for Reporting Implementation Studies (StaRI) [22].

### 2.1. Participants

Recruitment for the overarching cluster randomized trial occurred in two waves. To be eligible centers had to have at least one classroom for 3–4-year-olds, a quality rating (i.e., evaluation of staff training and program standards) from the North Carolina Division of Child Development and Early Education of 3–5 stars (on a 5-star scale) or exempt from quality rating (e.g., faith-based organizations), provide lunch, not serve only children with special needs, and consent provided from at least one teacher and seven parents of 3–4-year-olds [17]. Only centers in the second wave of the trial were eligible for participation in the current study. This second wave included 51 ECE centers from rural and suburban areas in central North Carolina. The 29 ECE centers randomized to deliver HMHW during the 2016–2017 school year were designated standard implementation and served as a historical control. The 22 ECE centers randomized to the delayed intervention group were eligible to participate in the enhanced implementation during the 2017–2018 school year. Upon completion of the main trial, ECE center directors from the delayed intervention group were contacted via phone and e-mail about the opportunity to participate in this follow-up study regarding an enhanced implementation strategy. Thirteen of the 22 eligible centers (59%) consented to participate. Directors and teachers (i.e., providers) signed informed consent. Only parents who completed and returned an anonymous survey were considered to have consented to participate in this research study.

### 2.2. Intervention and Implementation Approaches

The HMHW intervention has been described at length [16,18]. Briefly, HMHW included kick-off and celebration events as well as four, 6-week units of branded, complementary educational materials (e.g., Family Guides, Activity Trackers) and interactive activities for use in the classroom and at home. To deliver the ECE center-based portion of HMHW, ECE providers received implementation support from the research team through educational manuals, classroom resources, two interactive educational meetings, and centralized technical assistance at three points during the intervention—before the kick-off event and near completion of units 1 and 3 [16]. In turn, ECE providers were expected to provide implementation support to parents for the home-based portion of HMHW by providing intervention materials, prompting participation at home by sending reminder Our Turn cards, and inviting parents (e.g., flyers and e-mail) to participate in events at the ECE center. Development of the standard approach has been previously described at length and is summarized in Table 1 [16,18,19].

To develop the enhanced implementation approach, we conducted a mixed methods comparative case study among seven centers from Wave 1 of the overarching trial that demonstrated low and high parent involvement with HMHW [23]. Two frameworks informed the development of semi-structured interview guides and the initial coding frameworks. The Consolidated Framework for Implementation Research [24] provided a structure for exploring determinants (i.e., barriers and facilitators) that influenced implementation of HMHW. Epstein’s framework for school–family–community partnerships provided practical guidance for developing partnerships between educational organizations and families [25]. In applying both frameworks, we identified contextual and practical elements influencing parent involvement with the intervention. Notably, ECE providers rarely inquired about parents’ experiences with the program at home. As such, we identified (1) ‘intervening with parents to enhance uptake and adherence’ and (2) ‘obtaining and using parents’ feedback’ as two promising strategies for ECE providers to solicit feedback about parents’ experiences at home and to help identify and address barriers to participation (described in Table 1) [26,27,28].

**Table 1:** Discrete implementation strategies [28] selected for early care and education providers (actors) to use with parents of 3–4-year-old children (target) to support adoption and implementation of the *Healthy Me*, *Healthy We* intervention.

Implementation Strategy [28]	Action	Target	Temporality and Dose	Implementation Outcome [20]	Justification
**Standard**					
*Distribute educational materials*	Teachers distribute and explain family guides to parents in person	Parents’ knowledge about the program and targeted behaviors. Resources and opportunities for parents to practice targeted behaviors	At the start of each unit Four units	Adoption Fidelity to program activities	Family guides were key source of information to guide parents through program participation
*Remind families*	Teachers distribute *Our Turn* cards to parents in person	Prompt families to do program activities at home	Send home the same day a classroom activity is completed At least 32 times: eight or more times during each of the four units	Fidelity to program activities	Prompts/cues and reminder systems have been shown to promote adherence and engagement [29]
*Involve parents or other family members*	Directors and teachers invite parents to attend or otherwise support (e.g., sending food for tasting events) kick-off, celebration, or other classroom activities	Knowledge about the intervention, sharing child’s excitement, and realizing effect of intervention will increase parents’ understanding of their role and motivation to implement program at home	During the 8-month intervention period At least twice (for kick-off and celebration events)	Adoption Fidelity to program activities	Knowledge and skills for new practices or programs can support buy-in and participation [30]
**Enhanced (standard strategies, plus the following)**					
*Intervene with parents to enhance uptake and adherence*	Directors and teachers use conversation starter cards to initiate communication about classroom and home activities or general eating and physical activity behaviors, which may include providing encouragement, role modeling behaviors and activities, and/or problem solving	Identify and address barriers parents face completing home activities and/or targeted behaviors to promote healthier eating and physical activity (BCT 1.2: problem solving)	Initiate before prompting parents to do a home activityAt least eight times: two or more times during each of the four units	Acceptability Appropriateness Adoption Feasibility Fidelity to program activities	Problem solving identified barriers can promote program adoption and continued engagement [26,27]
*Obtain and use parents’ feedback*	Directors and teachers use follow-up conversation starter cards to initiate communication about experiences with home and classroom activities to evaluate what could be done differently in the delivery or support to deliver the intervention at home or within classroom	Create (more) explicit opportunities to solicit and act on feedback about the program and targeted behaviors (BCT 1.6: discrepancy between current behavior and goal)	Initiate within 1 week after prompting parents to do a home activity At least eight times: two or more times during each of the four units	Acceptability Appropriateness Adoption Feasibility Fidelity to program activities	Feedback can identify whether approach is working and either reinforce efforts or determine how to improve or approach differently [27]

BCT indicates behavior change technique [31].

### 2.3. Data Collection Procedures and Measures

Data collection occurred at five time points: baseline, during the intervention at 2, 4, and 6 months, and immediately following the 8-month intervention period. Measures included a series of observations, structured interview questions, and self-reported surveys. ECE providers received $15 compensation for completing all study measures. Due to the anonymous nature of surveys, parents were not offered compensation.

#### 2.3.1. Demographics

ECE providers and parents self-reported age, race, ethnicity, sex, and education. Parents also reported marital status and providers reported the length of time working in their current position at the ECE center. Center directors provided information about their ECE center by indicating yes or no regarding their center’s accreditation by the National Association for the Education of Young Children; acceptance of child care subsidies; participation in the federally-funded Child and Adult Care Food Program; type of ECE program; and utilization of healthy living curricula. Directors also provided the number of children attending the center and weekly enrollment fees. Additionally, prior to the intervention, trained and blinded data collectors completed the document review component of the Environment and Policy Assessment and Observation tool [32]. This tool identifies evidence of policies pertaining to parent engagement around nutrition, physical activity, screen time, and outdoor play and learning. The standard group completed demographic measures during the 2016–2017 school year. The enhanced implementation group completed measures during the 2017–2018 school year.

#### 2.3.2. Parent Engagement

The primary outcome of parent engagement was evaluated in two ways: (1) a center-level indicator and (2) parent-level indicator. The center-level measure of parent engagement (Table 2) compiles input from directors and teachers into a single, unweighted score representative of providers’ perceptions of parent engagement for the duration of the intervention. The research team conducted structured interviews with directors (total of 9 items) and teachers (total of 6 items) during two of the technical assistance visits, near completion of units 1 and 3 (~2 and 6 months into the intervention). Interview questions focused on implementation of the program (e.g., difficulty implementing the program), communication with parents about HMHW (e.g., methods for distributing materials; difficulty communicating), and ECE providers’ perceptions of family participation at home (e.g., evidence of children doing activities at home; receiving feedback from families about HMHW). Due to the multi-level nature of this intervention, implementation in the classroom was included as a center-level measure of parent engagement under the presumption that teacher behaviors related to HMHW in the classroom, or the lack thereof, would influence opportunities for parents to be involved.

The qualitative interview responses were translated to quantitative values through a systematic scoring process that applied two- (e.g., yes/no) or three-point (e.g., none/some/a lot) scales for each interview question. To enhance rigor, two members of the research team, blinded to the cohort assignment, individually coded responses and met to resolve discrepancies and determine final scores. Values were then summed, unweighted, to generate a total score (range 0–27). Higher scores indicated fewer barriers and more parent engagement.

The parent-level measure was based on surveys completed by parents (20 items) regarding participation in kick-off and celebration events, understanding of the intervention, receipt of intervention materials, and use of materials and participation at home. Surveys were completed at the end of the intervention period (Table 3). Individual parent scores (range 0–45) were generated by summing all items. Items were scored using two- (e.g., disagree/neutral or agree), three- (e.g., yes/no/unsure), or five-point (i.e., strongly disagree to strongly agree) scales. Higher scores indicated parents participated in events and had received and used intervention materials. To assess construct validity, parents in the enhanced group completed the short form of the Family and Provider/Teacher Relationship Quality (FPTRQ) [33]. The FPTRQ is a previously validated measure of the quality of relationships between parents and ECE providers of children birth to 5 years of age [34]. It evaluates professional practices, attitudes, and knowledge about individual families that are theoretically relevant to parent engagement. Parent-level scores of parent engagement in the enhanced group demonstrated a medium–large statistically significant positive association with the FPTRQ parent total scores (r(104) = 0.46, *p* < 0.0001).

#### 2.3.3. Implementation Outcomes for the Enhanced Implementation Approach

After the intervention period, ECE providers and parents in the enhanced group completed a 7-item survey regarding the acceptability, appropriateness, and feasibility of the enhanced implementation approach for HMHW [20,35]. Fidelity to the enhanced implementation approach was evaluated via self-report surveys from ECE providers at the midpoint (4 months) and completion (8 months) of the intervention. Survey items asked about initiation of specific implementation strategies (e.g., Have you asked parents for feedback about their experience with the Healthy Me, Healthy We program?) and adherence to specified dose (e.g., Since September, how often have you met with or talked to parents about their experience with or questions about doing the Healthy Me, Healthy We program at home?) [36].

### 2.4. Statistical Analyses

Demographic characteristics and implementation outcomes were summarized with descriptive statistics, including frequencies, proportions, means, and standard deviations. Chi-square tests and Fisher’s exact tests were used to assess differences between groups. Two-sample *t*-tests and Wilcoxon rank tests were used to evaluate differences for continuous data. The difference between groups for the center-level indicator of parent engagement was evaluated with a linear regression model. The model included covariates for having at least one policy regarding parent engagement with health promotion (identified a priori), as policies may implicate differing baseline levels of parent engagement with health promotion across centers, and to control for statistically significant differences between cohorts regarding the number of years providers had worked at ECE centers. Given the sample size of centers in each group and desiring a two-sided test of significance with α = 0.05 and β = 0.2, there was power to detect a standardized mean difference of 0.91. The difference between groups for the parent-level indicator of parent engagement was evaluated with a mixed effect model that included a random intercept to account for clustering of parents within ECE centers. The model included sex, race and ethnicity, education, and marital status as covariates. The ICC estimate from the model was 0.17. Effect sizes were estimated with Cohen’s d (small 0.2; medium 0.5; large 0.8) [37]. Statistical significance was set at *p* < 0.025 to account for two primary outcomes. All statistical analyses were performed in SAS version 9.4 (SAS Institute, Inc., Cary, NC, USA).

## 3. Results

This study involved 42 ECE centers—29 centers in the standard implementation group and 13 centers in the enhanced implementation group. Both groups similarly represented faith-based, pre-kindergarten, and Head Start programs, were comparable regarding the number of children enrolled and weekly enrollment fees, and a majority accepted child care subsidies and participated in the Child and Adult Care Food Program (Table 4). The demographic characteristics of ECE centers that consented to participate in the enhanced implementation group were not different from eligible centers that opted not to participate in the enhanced implementation or Wave 2 of the larger randomized trial (all *p* > 0.10).

A total of 173 ECE providers and 313 parents participated in this study (Table 5). ECE providers in both groups predominately identified as female. About half of the providers identified as non-Hispanic Black and less than half reported obtaining a college or graduate degree. Groups were similar, except providers in the enhanced group had worked at their centers longer than providers in the standard group. Parents mostly identified as female and either non-Hispanic White or non-Hispanic Black. About half (58%) reported they were married and had a college or graduate degree (47%). There were no statistically significant differences between the groups.

The mean center-level parent engagement score was 14.1 ± 3.7 for the standard implementation group and 13.2 ± 5.7 for the enhanced implementation group (possible range: 0–27). The unadjusted model for the center-level indicator showed no difference in parent engagement scores (*p* = 0.51) between groups, with the small effect size (*d* = −0.21) favoring the standard implementation group. The adjusted model, which controlled for having at least one policy regarding parent engagement with health promotion and number of years providers had worked at ECE centers, also showed there was no difference in the effect of the type of implementation approach, *β* = −1.45 (95% confidence interval, −4.76 to 1.87), *p* = 0.38.

The mean parent-level parent engagement score was 21.4 ± 8.6 for the standard implementation group and 25.1 ± 8.7 for the enhanced implementation group (possible range: 0–45). The unadjusted model for the parent-level indicator showed a difference in parent engagement scores (*p* < 0.001) between standard and enhanced implementation. The small–medium effect size (*d* = 0.42) favored the enhanced implementation group. The adjusted model, which controlled for parent sex, race and ethnicity, education, and marital status, also showed the enhanced group had higher parent-level scores of parent engagement compared to the standard group, *β* = 3.60, (95% confidence interval, 1.49 to 5.75), *p* < 0.001.

**Figure 1:**
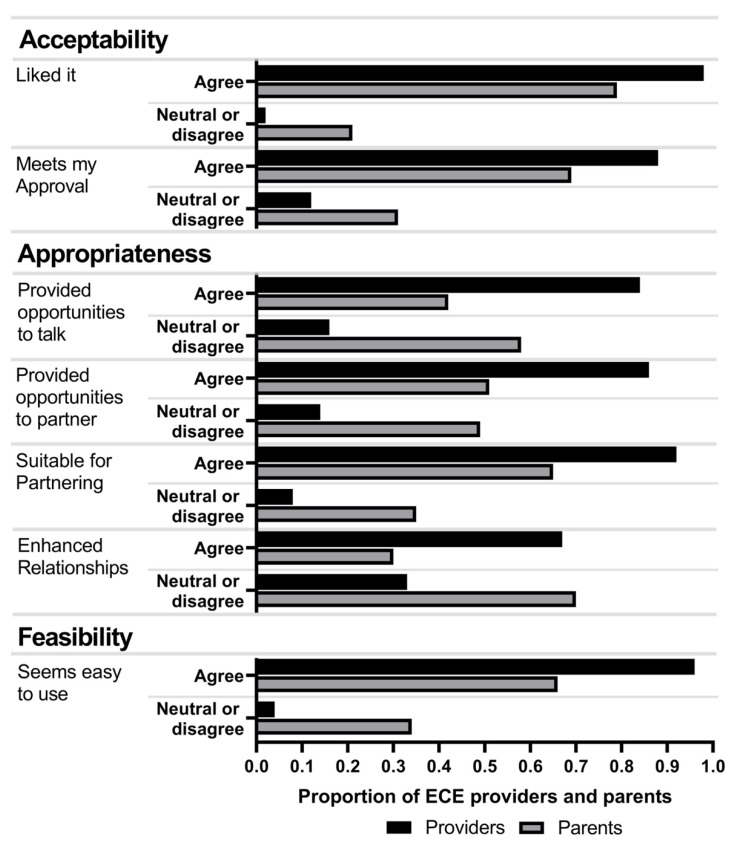
Acceptability, appropriateness, and feasibility ratings of *Healthy Me*, *Healthy We* by early care and education (ECE) providers (n = 57) and parents (n = 114) in the enhanced implementation group. Differences in proportion of responses by providers and parents all statistically significant at *p* < 0.001.

Within the enhanced group, there was a significant relationship, *p* < 0.001, between caregiver role and perceived acceptability, appropriateness, and feasibility of the HMHW intervention (Figure 1). ECE providers consistently reported more favorable views than parents regarding satisfaction with the intervention for partnering and encouraging healthy eating and physical activity, the practicality and suitability for partnering to encourage healthy eating and physical activity, and the ease with which the HMHW intervention could be used. Fidelity to the package of strategies for the enhanced implementation approach, however, was low (less than 20%) (Table 6). Most ECE providers (50–92%) reported carrying out discrete implementation strategies at least once by the midpoint of the intervention period and at least once in the second half of the intervention (48–85%). However, adherence to the prescribed dose of each strategy for the duration of the intervention was consistently low (28–46%), except for distribution of educational materials (81%).

## 4. Discussion

This study is one of the first to investigate the efficacy of implementation strategies to increase parent involvement with health promotion interventions in ECE [13]. Many innovations aim to involve parents, but few have included adequate implementation support [10]. The small increase in parent engagement observed among parents who received an enhanced implementation approach, despite ECE providers’ relatively low fidelity to this approach, shows promise for the capacity to increase parent involvement with health promotion efforts through ECE and may suggest that the quality and quantity of strategies or interactions between ECE providers and parents is important [38]. Engaging parents during the early childhood period is critical but challenging [15], and there is a need for continued investigation to identify effective strategies and to better characterize these strategies [39].

Similar to other studies, ECE providers expressed generally positive opinions about the acceptability and feasibility of the intervention [40,41,42]. This study extended the work of others by seeking parents’ opinions which, interestingly, were less favorable than ECE providers’ opinions. Implementation outcomes can serve as indicators of implementation processes [20], and differences in opinions between ECE providers and parents could be a marker that parents did not receive adequate implementation support for participation. In turn, differences in perceptions or lack of communication between ECE providers and parents regarding their roles with specific interventions, or health promotion at large, could negatively influence their ability to partner and ultimately influence implementation [40]. It is important to ensure ECE providers receive support to successfully involve parents in implementation efforts. Traditionally, training and technical assistance have been effective for center-level innovations (e.g., policies) [43]. However, these results highlight the complexities of providing adequate implementation support for multiple levels of intervention occurring in community-based settings like ECE centers and suggest different or additional content and strategies are needed to enable ECE providers to involve parents in implementation efforts [44].

Fidelity to intervention and implementation processes is critical for achieving desired outcomes [45]. Yet, few studies measure and report the extent to which interventions are delivered as intended in ECE settings [46]. Even fewer report fidelity of implementation strategies. When reported, fidelity to individual components as well as the intervention at-large widely vary, which may be related to the complexity of the intervention or implementation design. In this study, a large proportion of ECE providers reported trying individual implementation strategies, but adherence to the dose of the enhanced implementation approach for HMHW was quite low. The strategy with the highest fidelity (distributing educational materials) likely aligned with providers’ workflow and did not require a change in routine. Other factors (e.g., structure and function of ECE program or characteristics of ECE providers) may have influenced uptake of more dynamic strategies (e.g., helping parents identify and address barriers), and additional support may be needed to integrate into providers’ routine [24]. Measuring fidelity and applying findings throughout the intervention period could identify potential issues and solutions regarding inadequate implementation and reduce the opportunity for “Type III errors” or observing a null effect for a program that has not been adequately implemented [47].

Results also highlight the complexities of providing adequate implementation support for multiple levels of interventions which occur in community-based settings like ECE centers and the importance of designing interventions with implementation in mind [44]. Although strategies were selected to address previously identified barriers [23], it is worth considering whether the strategies were in fact the best match for ECE providers to employ with parents. Future planning and implementation efforts should involve all stakeholders in the strategic selection, specification, tailoring of implementation strategies [48,49]. This could lead to strategies that are feasible within the context of ECE settings, acceptable for both ECE providers and parents, and potentially improve fidelity and subsequent outcomes [7].

Previous efforts to measure parent engagement have been limited [13,14] and lack the assessment of dynamic processes between ECE providers and parents [15]. This study employed multiple measures of parent engagement that included input from both ECE providers and parents. The mixed results from measuring parent engagement at multiple levels (i.e., center-level and parent-level) may be due to different measurement approaches, but importantly indicate parent engagement and factors that influence it need to be evaluated comprehensively. Within the Head Start model for ECE, there have been recent distinctions between parent involvement and engagement [50]. ‘Parent involvement’ refers to parents participating in activities and taking advantage of opportunities at the ECE center, while ‘family engagement’ refers to interactive relationship-building processes between ECE providers and parents or other caregivers. This spectrum introduces a menu of options to target different levels of involvement or engagement to meet the needs of both ECE providers and parents, and it recognizes relationship building and trust as foundational elements. Future efforts should explore the applicability of the model for ECE health promotion programs, evaluate the influence of the underlying context of relationships and trust between providers and parents, and measure parent engagement as dynamic, ongoing interactions between ECE providers and parents, rather than a static event [15].

This study had several strengths including being one of the first to explicitly target parent engagement as an implementation strategy for supporting health promotion efforts in ECE centers, applying multiple frameworks to select implementation strategies to address identified barriers, and extending the conceptualization and measurement of parent engagement to capture perspectives of both ECE providers and parents. Despite these strengths, there were several limitations. First, the quasi-experimental nature of this study, including use of a historical control group, limits the inference of causality regarding the effect of the enhanced implementation strategy on parent engagement. The self-selection of ECE centers and parents to participate in this study may have introduced bias to the study results. However, this study design is useful when there are practical barriers to conducting a randomized experiment, such as that experienced when capitalizing on delayed intervention groups as an opportunity to refine intervention and implementation efforts, and to gain evidence to support future investigation [51]. Second, the generalizability of this study is limited. It was not possible to compare characteristics of participating families to those who did not. Parents who participated in this study are not necessarily representative of those with lower levels of education or more diverse family structures. Additionally, there may be unmeasured characteristics of ECE centers, providers, or parents that differ between groups, thus potentially limiting the internal validity and generalizability of results. Finally, measures for parent engagement were created to evaluate parent engagement with HMHW and therefore lack generalizability and formal evaluation of reliability and validity. The assessment of parent-level engagement at the end of the intervention may have limited their ability to accurately recall engagement throughout, potentially over- or under-estimating their actual engagement. Although, the parent-level measure demonstrated a medium–large, statistically significant, positive relationship with an existing measure (FPTRQ).

## 5. Conclusions

This study demonstrated that an enhanced implementation approach can increase parent engagement with HMHW. Findings also identified important discrepancies between caregivers regarding acceptability and feasibility of the intervention and implementation approach, with ECE providers having more favorable opinions. Future efforts should strive to identify feasible and acceptable implementation strategies that can be implemented with fidelity for all stakeholders and to evaluate the effectiveness for increasing parent involvement and engagement with health promotion efforts. In turn, this evidence-base could be used to better understand what strategies work for whom and under what conditions, so that minimal burden is placed on those implementing programs and augment the impact. In summary, results show promise for future health promotion efforts in ECE in that parent involvement can positively respond to appropriately designed and executed implementation strategies. However, there is a great need to expand support for ECE providers to adequately implement innovations in ECE settings and include parents in those efforts. Developing and sustaining collaboration between ECE providers and parents will support implementation, accelerate translation, and support sustainability of these evidence-based interventions to yield longer-term benefits for young children.

## Figures and Tables

**Table 2:** Center-level measure and scoring of parent engagement for *Healthy Me*, *Healthy We* (HMHW) from structured interviews during technical assistance visits.

Items	Source	Time Point	Scoring
Perceived strength of doing HMHW is that it connects to home	Director	Unit 1	0: No 1: Yes
Difficult for teachers to execute program	Director	Unit 1	0: Yes 1: No
Difficulty with communication between teachers and parents or parent participation	Director	Unit 1	0: A lot 1: Some 2: None
Parent participation in kick-off	Director	Unit 1	0: No 1: Unsure 2: Yes
Received feedback from families	Director	Unit 1	0: None 1: Some 2: A lot
Difficult to execute program	Teacher	Unit 1	0: A lot 1: Some 2: None
Received feedback from families	Teacher	Unit 1	0: None 1: Some 2: A lot
Methods to hand out family materials	Teacher	Unit 1	0: Passive 1: Directly hand materials 2: Interact with families beyond handing materials out
Difficult for teachers to execute program	Director	Unit 3	0: A lot 1: Some 2: None
Difficulty or decrease with communication between teachers and parents or parent participation	Director	Unit 3	0: A lot 1: Some 2: None
Perceived change or strength of doing HMHW is that it connects to home	Director	Unit 3	0: No 1: Yes
Received feedback from families	Director	Unit 3	0: None 1: Some 2: A lot
Difficult to execute program	Teacher	Unit 3	0: A lot 1: Some : None
Difficulty or decrease with communication between teachers and parents or parent participation	Teacher	Unit 3	0: A lot 1: Some 2: None
Evidence of families doing activities at home	Teacher	Unit 3	0: None 1: Some 2: A lot or great increase

**Table 3:** Parent-level measure and scoring of parent engagement for *Healthy Me, Healthy We* from surveys completed after the intervention period.

Items	Scoring
***Materials Received (10 items)***	
Family Guide (Units 1–4)	0: No 1: Unsure 2: Yes
At Home Activity Tracker (Units 1–4)	0: No 1: Unsure 2: Yes
Our Turn Trading Cards	0: No 1: Unsure 2: Yes
Number of Our Turn Trading Cards	0: Unsure 1: 0–4 2: 5–9 3: 10–14 4: 15–24 5: 25 or more
***Center Participation (4 items)***	
Center hosted a kick-off or celebration event	0: No 1: Unsure 2: Yes
Parent participated in kick-off or celebration event	0: No 1: Yes
***Home Participation (6 items)***	
Number of activities tried at home	0: Unsure 1: 0–4 2: 5–9 3: 10–14 4: 15–24 5: 25 or more
Parent understands the program	0: Very poorly 1: Poorly 2: Adequately 3: Well 4: Very well
Parent understands what is being asked of them	0: Very poorly 1: Poorly 2: Adequately 3: Well 4: Very well
Parent read about half or more of Family Guides	0: Strongly disagree, disagree, neither agree or disagree 1: Agree or strongly agree
Parent tried the ‘Just Try It’ suggestions from the Family Guides	0: Strongly disagree, disagree, neither agree or disagree 1: Agree or strongly agree
Parent tried the recipes in the family guides	0: Strongly disagree, disagree, neither agree or disagree 1: Agree or strongly agree

**Table 4:** Characteristics of 42 early care and education centers implementing *Healthy Me*, *Healthy We*.

Criteria	Standard Implementation (n = 29)	Enhanced Implementation (n = 13)
Accredited by the National Association for the Education of Young Children, n (%)	10 (36)	4 (31)
Accepts child care subsidies, n (%)	24 (89)	12 (92)
Participates in the Child and Adult Care Food Program, n (%)	23 (79)	10 (77)
Other program affiliations, n (%) ^a^		
Faith-based	9 (31)	5 (38)
NC Pre-K or other pre-kindergarten	7 (24)	2 (15)
Head Start and/or Early Head Start	7 (24)	1 (8)
Use health promotion curricula, n (%)	11 (38)	4 (31)
At least one policy regarding parent engagement with health promotion, n (%)	17 (59)	4 (31)
Total child enrollment, mean (range)	90 (28–218)	82 (25–170)
Weekly enrollment fees for 3–4-year old children, mean	$129	$133

^a^ Could select all that apply.

**Table 5:** Demographic characteristics of early care and education providers (n = 173) and parents (n = 313) implementing *Healthy Me*, *Healthy We*.

	Standard Implementation		Enhanced Implementation	
Characteristics	Providers (n = 116)	Parents (n = 199)	Providers (n = 57)	Parents (n = 114)
Sex, female, n (%)	114 (98)	154 (80) *	55 (96)	100 (89) *
Age, years (mean ± sd)	41 ± 12.2	33 ± 7.6	41 ± 13.1	33 ± 7.5
Race and ethnicity, n (%)				
Non-Hispanic Black	60 (54)	78 (43) *	29 (51)	33 (31) *
Non-Hispanic White	34 (31)	85 (47)	23 (40)	58 (54)
Other ^a^	17 (15)	19 (10)	5 (9)	17 (16)
Highest level of education completed, n (%)				
Some college or lower	31 (27)	76 (40)	18 (32)	38 (34)
Associate degree	33 (28)	22 (12)	13 (23)	16 (14)
College degree or higher	52 (45)	90 (48)	26 (46)	57 (51)
Years in current position ^b^ (mean ± sd)	9 ± 8.5	-	7 ± 7.4	-
Years working at center ^b^ (mean ± sd)	5 ± 5.6 **	-	9 ± 8.8 **	-
Marital status, n (%) ^c^				
Married or domestic partnership	-	109 (58) *	-	74 (69) *
Not married	-	78 (42) *	-	34 (31) *

sd indicates standard deviation * *p* < 0.10; ** *p* < 0.001. Chi-square tests and Fisher’s exact tests used to evaluate difference in distribution, and two-sample *t*-tests used to compare means between providers and parents in the standard and enhanced implementation groups. ^a^ Other race and ethnicity includes American Indian/Alaska Native, Asian, Hispanic or Latino, and more than one race; ^b^ Years in current position and working at center were only measured for providers; ^c^ Marital status was only measured for parents.

**Table 6:** Early care and education providers’ fidelity to the enhanced implementation approach for *Healthy Me*, *Healthy We*.

Implementation Strategy	Adherencen n (%)
Distribute educational materials ^a^ (n = 39)	31 (81)
Remind families ^a^ (n = 39)	18 (46)
Involve parents or other family members (n = 54)	18 (33)
Intervene with parents to enhance uptake and adherence (n = 54)	15 (28)
Obtain and use parents’ feedback (n = 54)	25 (46)
All strategies	8 (15)

^a^ Strategy prescribed only for teachers.

## Data Availability

Data can be made available by contacting the corresponding author.
